# Vaccination with dendritic cells loaded with allogeneic brain tumor cells for recurrent malignant brain tumors induces a CD4^+^IL17^+^ response

**DOI:** 10.1186/2051-1426-2-4

**Published:** 2014-02-18

**Authors:** Michael R Olin, Walter Low, David H McKenna, Stephen J Haines, Tambra Dahlheimer, David Nascene, Michael P Gustafson, Allan B Dietz, H Brent Clark, Wei Chen, Bruce Blazar, John R Ohlfest, Christopher Moertel

**Affiliations:** 1 Department of Pediatrics and the Masonic Cancer Center, University of Minnesota, 3-136 CCRB, 2231 6th St SE, Minneapolis, MN 55455, USA; 2 Department of Neurosurgery, University of Minnesota, 2001 6th St SE, Rm 4-216, Minneapolis, MN 55455, USA; 3 Department of Laboratory Medicine and Pathology, University of Minnesota, 8609B, 420 Delaware St SE, Minneapolis, MN 55455, USA; 4 Department of Radiology, University of Minnesota, 8292A, 420 Delaware St SE, Minneapolis, MN 55455, USA; 5 Department of Laboratory Medicine and Pathology, 200 First St, Mayo Clinic, Rochester, MN 55901, USA

**Keywords:** Vaccine, Brain tumor, Dendritic cell, Immunotherapy

## Abstract

**Background:**

We tested the hypothesis that a novel vaccine developed from autologous dendritic cells (DC) loaded with cells from a unique allogeneic brain tumor cell line (GBM6-AD) would be well-tolerated and would generate an immune response.

**Method:**

Patients with recurrent primary brain tumors underwent vaccination with GBM6-AD/DC vaccine. Subjects were treated at escalating DC cell doses: 5 × 10^6^ (one patient), 10 × 10^6^ (one patient) and 15 × 10^6^ (6 patients). Subcutaneous injections were planned for days 0, 14, 28, 42, 56, and monthly thereafter. The primary endpoint was the safety of the GBM6-AD/DC vaccination. The secondary endpoints were immune response, measured by flow cytometry, and the clinical outcome of tumor response defined by time to progression and overall survival.

**Results:**

Eight patients were treated. The first three patients were treated in the dose escalation phase of the trial; the remaining five patients received the maximum dose of 15 × 10^6^ DC. No dose limiting toxicity was observed. The best response per modified McDonald criteria was partial response in one patient. Flow cytometric immune profiling revealed significant differences in CD4^+^IL17^+^ lymphocytes and myeloid derived suppressor cell populations between patients characterized as having stable vs. non-stable disease.

**Conclusion:**

This first-in-human study shows that the GBM6-AD/DC vaccine was well tolerated and was associated with an immune response in a subset of patients. No MTD was achieved in this trial. This small-scale pilot provides information for larger scale investigations into the use of this allogeneic vaccine source.

## Background

Primary brain tumors continue to represent a significant therapeutic challenge. Current standard therapy employing radiation and temozolomide for WHO grade IV astrocytoma (glioblastoma multiforme) has resulted in extending the median survival from 12.1 months to 15 months [1,2], with greater than 85% of the patients dying within 3 years [3,4]. Likewise, recurrent pediatric tumors such as infratentorial ependymoma and medulloblastoma continue to have poor prognoses.

Given the lack of progress with current therapy, increased attention has been placed on therapeutic vaccination as a route to improved disease outcome and quality of life for these patients. Autologous tumor cells are frequently used as the source of vaccine antigen, functioning as personalized immunotherapy by targeting multiple patient-specific tumor antigens [5,6]. Vaccines utilizing tumor cells include tumor lysate-pulsed DCs [7-9], tumor-derived heat shock proteins [10], and whole tumor cell lysates [11-14]. A recent search revealed 25 clinical trials utilizing autologous vaccine for both high- and low-grade glioma (*Clinicaltrials.gov*). However, the use of patients’ resected tumors has a number of drawbacks and limitations. Autologous tumor-based vaccines require the removal and processing of the tumor to derive lysate. Although this process may result in an abundant amount of material, the yield of tumor cells may be low. In addition, contamination with impurities such as non-tumor derived endothelial cells, fibroblasts, leukocytes or other stromal cell types may occur. In some trials, resected tumors are cultured to purify tumor cells. However, the ability to expand the culture varies from tumor to tumor, potentially resulting in a lack of material for continued treatment. Moreover, if tumor resection is not an option, the patient is ineligible for treatment.

In this study, we used autologous dendritic cells (DCs) loaded with apoptotic bodies derived from an allogeneic glioma cell line (GBM6-AD) with a high expression of multiple tumor and tumor-associated antigens. To enhance immunogenicity, we incubated the cell line in physiologic (5%) oxygen, which we have previously reported to produce superior immunogenicity compared to cell lines grown under atmospheric (20%) oxygen conditions [11,15]. The purpose of this clinical trial was to evaluate the feasibility and toxicity of a novel DC vaccine loaded with antigen derived from our tumor cell line [15]. Our data suggest that this vaccine approach, using an allogeneic antigen source, is safe and induces potentially therapeutic immune responses in a subset of patients. In addition, by measuring various immune parameters, we discovered an increase in production of cytokine IL17a in patients demonstrating response to therapy.

## Results and Discussion

### Patient characteristics

Twelve patients were enrolled for this single institution study. All underwent apheresis. 2 patients withdrew due to rapid disease progression, 1 patient withdrew voluntarily, and 1 patient was declared ineligible due to the diagnosis of supratentorial PNET upon pathology review. Of the 8 patients who received vaccine treatment, one patient was not evaluable after having received only a single vaccine due to post-operative complications. All of the patients had received at least one prior treatment regimen containing temozolomide. 2 patients received prior bevacizumab; one of these patients also received cis-retinoic acid. We also made an effort to create a minimal residual disease state when possible, performing debulking surgeries in three subjects (patients 2, 3 and 8) prior to initiating vaccine therapy. All subjects were off dexamethasone therapy at the time of study enrollment.

At study entry, the mean absolute white blood cell count was 5,037 × 10^9^/L (range: 3,600 - 13,000 × 10^9^**/**L) and the mean absolute lymphocyte count was 1,162 × 10^9^/L (range 500 – 2,100 × 10^9^**/**L).

### Dose escalation and toxicity

No MTD was reached. The only non-DLT toxicities observed were fatigue (patient 7) and skin reaction at the vaccine injection site. The maximum area of localized erythema and induration observed at the injection site was 3 × 7 cm (patient 7). Patient 1 had deep venous thrombosis prior to vaccine administration that continued throughout his treatment course. No systemic reactions or anaphylaxis episodes were noted throughout the trial.

### Response evaluation

**Figure 1:**
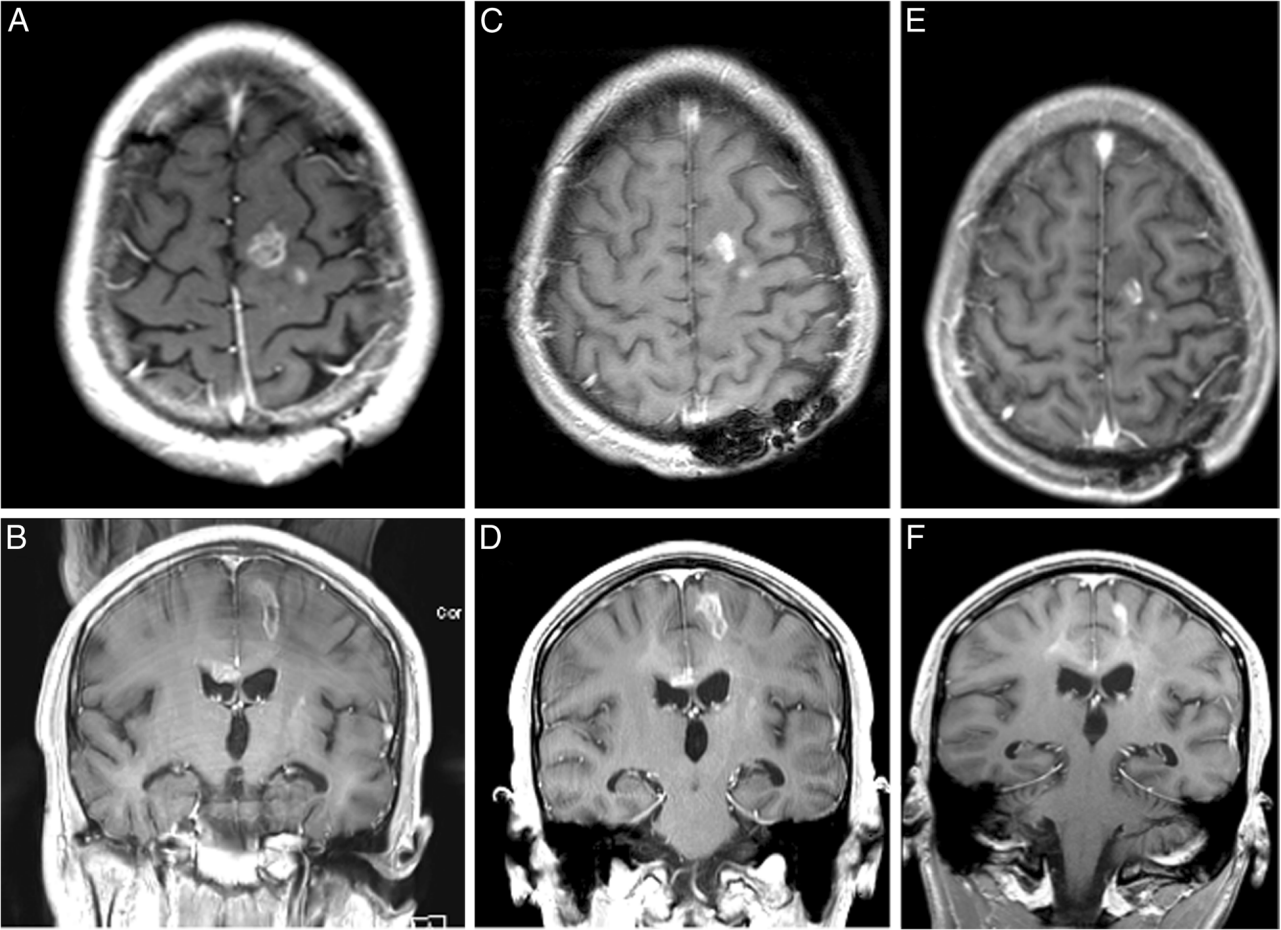
**Partial response in-patient following vaccination.** Axial and coronal MR images of a 17-year-old male with recurrent glioblastoma multiforme (patient 5) showing a partial radiographic response. Images show his scans at initiation of therapy **(A, B)**, at week 8 **(C, D)** and week 20 **(E, F)**.

The median number of vaccines administered was 4.5 (range, 1–12). Patient 4 had received bevacizumab prior to study entry. She met criteria for progression at week 13, but survived to 64.8 weeks after first vaccine. Patient 5 demonstrated multifocal tumor involvement at study entry, had a partial response by week 8 (Figure 1), and then failed distantly from his primary tumor sites at 32 weeks from first vaccine. Patients 5, 6 and 7 had stable disease for 30.6, 39.7 and 20 weeks respectively (Table 1). Patient 6 did not meet dimensional measurement criteria for progression, but had changes on perfusion imaging that led to biopsy and documentation of progressive disease. Hence, his therapy was terminated. Patient 7, with posterior fossa ependymoma, experienced recurrence at two distant intradural extramedullary spinal metastatic sites at thoracic spine levels 6 to 8 and lumbar vertebral levels 4 to 5. The remaining 7 of 8 evaluable patients experienced progression at their primary tumor site. Two patients (patient 6 and 7) remain alive off protocol therapy; patient 6 received metronomic temozolomide and is currently receiving bevacizumab 92 weeks from first vaccine and patient 8 received sunitinib and is 72 weeks from first vaccine. Patient groups were defined in a post hoc analysis as stable if they exhibited no progression at week 8 of vaccine therapy and non-stable if they showed progression at or before week 8.

**Table 1:** Vaccine release criteria was determined by pathogen load and phenotypic markers

**Assay**	**Test method**	**Result (median, range)**	**Specification**
Viability (AO/Pl)	Fluorescence microscopy	87.0% (61.0%-94.0%)	>70%
HLA-DR	Flow cytometry	94.5% (69.5%-98.4%)	>70%
CD86	Flow cytometry	87.0% (61.5%-94.8%)	>70%
Endotoxin	LAL	<0.7 (<0.4-1.030)	<5 EU/mL
Gram stain	Standard	No organisms	No organisms
Sterility (14 days)	Bactec	No growth	No growth
Mycoplasma	PTC (28 day culture)	Negative	Negative

### Patient evaluation

To determine if the immune response correlates with clinical observations, we divided patients into stable and non-stable populations based on time on trial and immune response (see Table 1).

### Phenotypic biomarkers

**Figure 2:**
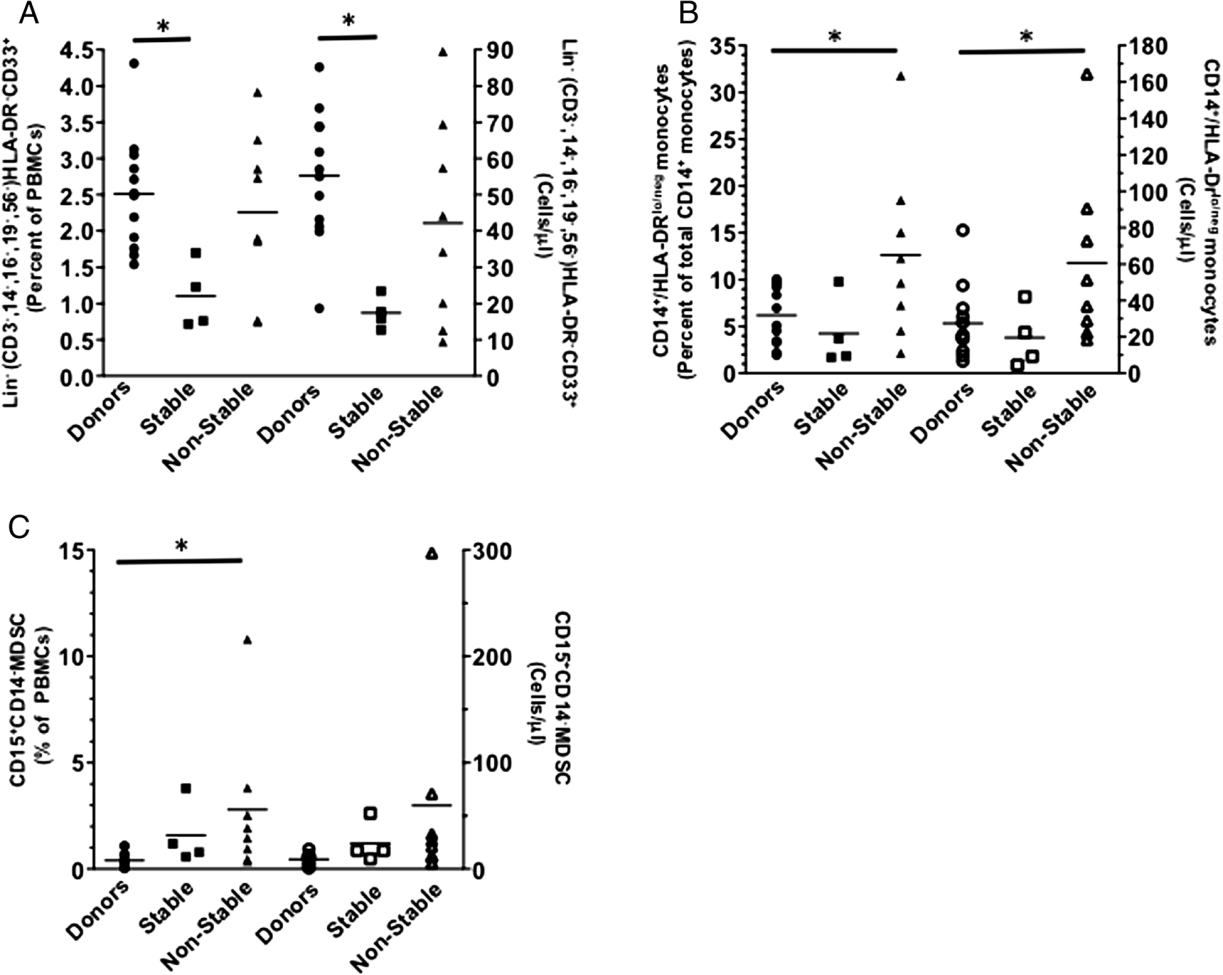
**Non-stable patients have higher MDSC populations.** Patients’ whole blood was stained with antibodies to analyze **A**. MDSC (Lineage negative), **B**. monocytic MDSC, and **C**. granulocytic MDSC populations from patients prior to vaccination. Error bars, ± SEM. *P < 0.05.

Myeloid-derived suppressor cells (MDSCs) represent a heterogeneous population of myeloid progenitor cells and immature myeloid cells. MDSCs suppress T cell function through the production of arginase-1, nitric oxide and reactive oxygen species [16]. Elevated numbers of MDSCs in peripheral blood have been demonstrated in a substantial number of studies in different types of cancer, including malignant glioma [17], head and neck cancer [18,19], invasive breast carcinoma [19], colon carcinoma [19] and mesothelioma [20]. The data presented in this manuscript demonstrate the same trend; we focused on the clinically stable and non-stable populations. In this study, patients 4, 5, 6 and 7, who were characterized with stable disease, had a statistically significant decrease in Lineage^neg^ (CD3^-^CD14^-^CD16^-^CD19^-^CD56^-^) HLA-DR^-^CD33^+^ MDSCs (Figure 2A) and monocytic MDSCs (CD14^+^HLA-DR^lo^) (Figure 2B). We did observe a statistically significant increase in the percentage of granulocytic MDSC (CD15^+^CD14^-^) (Figure 2C) in patients with non-stable vs. stable disease. The increased absolute number of granulocytic MDSCs in the non-stable group failed to reach statistical significance. No difference in Tregs was observed between patient populations (data not shown).

To investigate the use of phenotypic biomarkers as a tool to follow response to immunotherapy, we followed the development of effector and central memory CD4^+^ and CD8^+^ T cell populations throughout each patient’s time on the trial. CD4^+^ and CD8^+^ central memory populations sharply decreased and CD4^+^ and CD8^+^ effector memory populations increased by week 24 in our stable patient group (Additional file 1: Figures S1A-H). These observations failed to meet statistical significance between stable and non-stable groups. However, there was a significant increase of CD8^+^ central memory T cells (p = 0.037) in the stable disease population from week 0 to 24.

**Figure 3:**
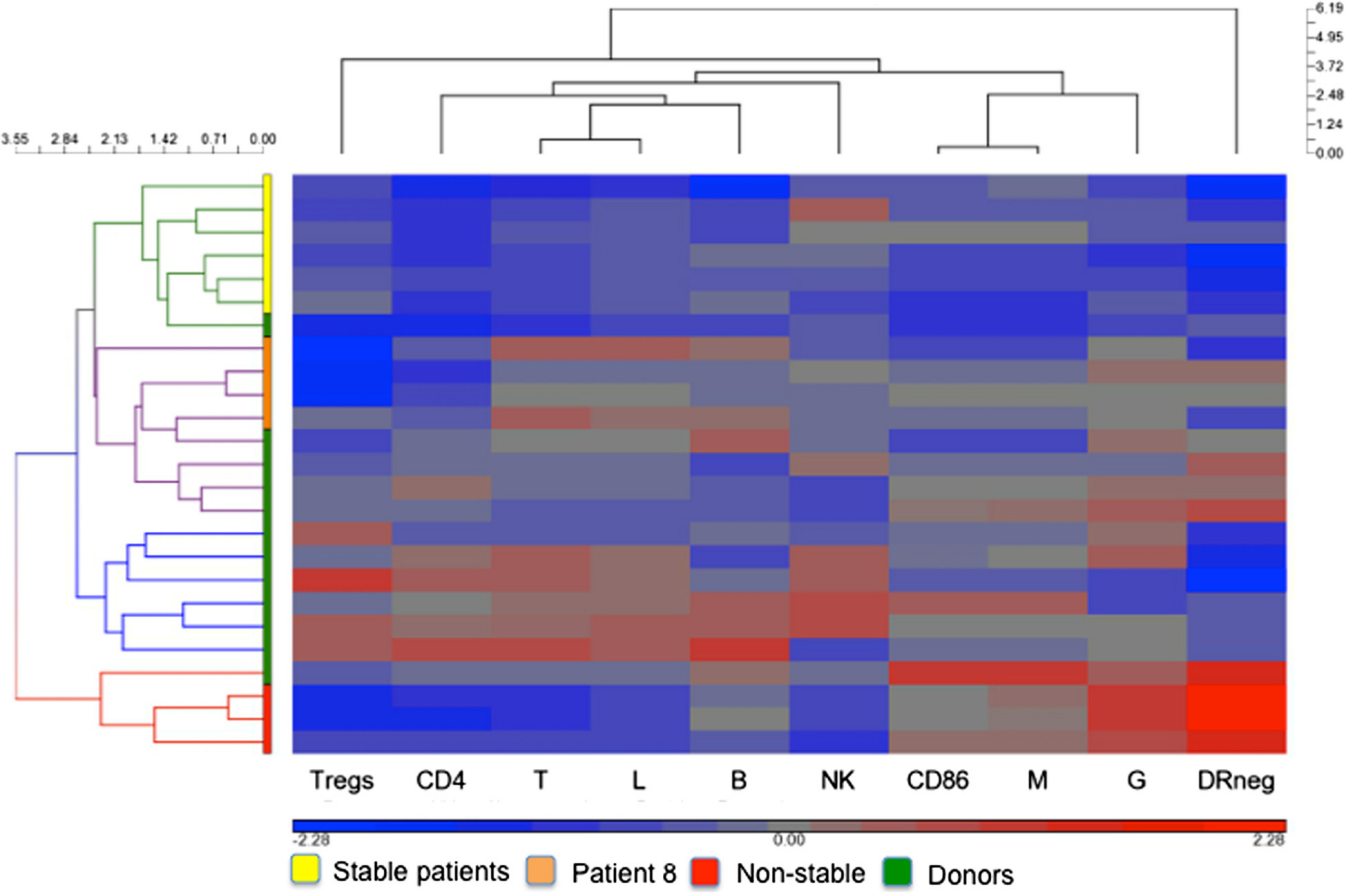
**Hierarchical clustering of immune phenotypes validates predicted stable and non-stable patient populations.** Phenotypic values were normalized to healthy donors and imported into genomic software for hierarchical clustering.

To test our predictions regarding the stable and non-stable patient populations, phenotypic markers from the patients were blindly normalized from healthy donors and imported into Partek Genomics software for hierarchical analysis as described by Gustafson et al. [21]. This categorized the same patients into the same groups that we predicted to be stable or non-stable populations (Figure 3) with the exception of patient 8, who was placed in the non-stable group based on her time on trial as a criterion. Using cluster analysis, the program placed her more closely immunologically to the stable patient population than the non-stable population. Interestingly, although she went off trial early due to progressive disease, patient 8 remains alive with disease.

### Cytokine production

**Figure 4:**
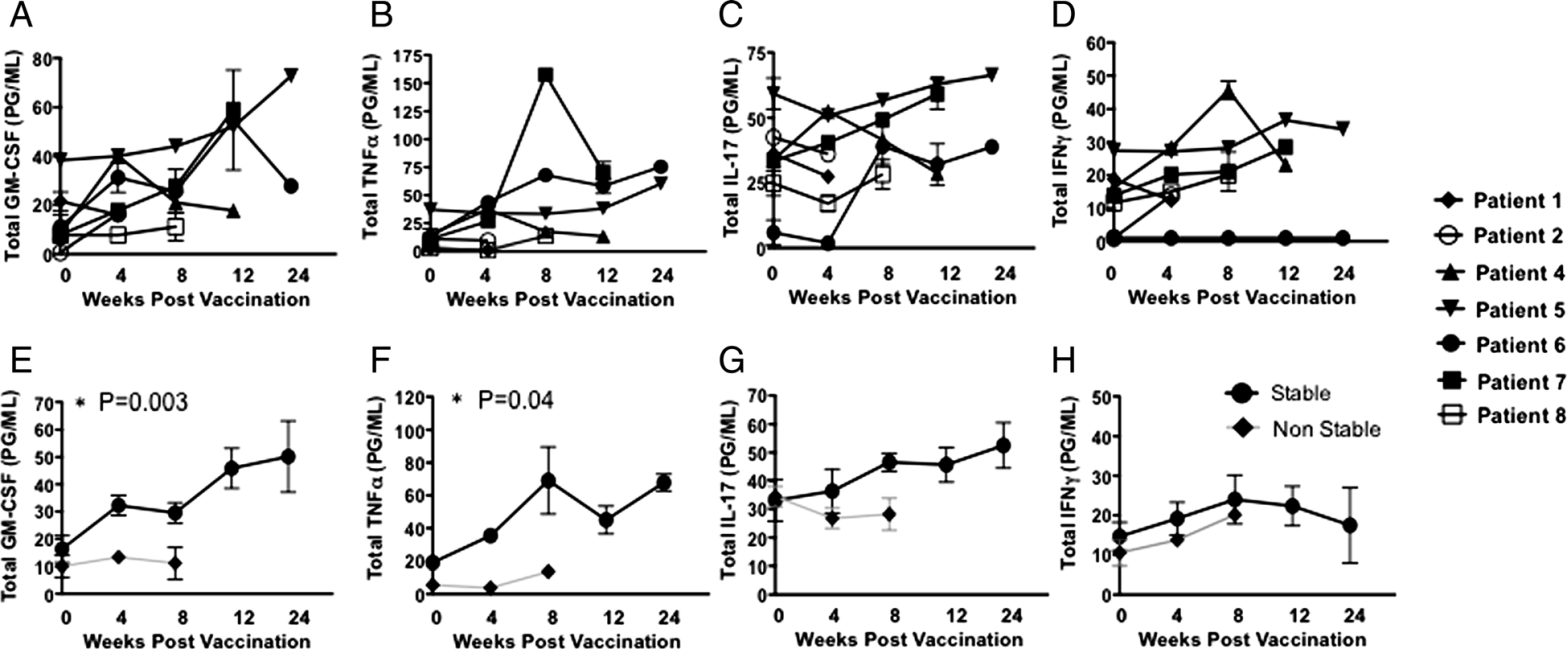
**Patients’ cytokine profiles are altered following vaccination.** Patients’ PBMCs were stimulated with tumor lysate-pulsed dendritic cells, maturated and analyzed for **A**. GM-CSF, **B**. TNFα, **C**. IL-17a, and **D**. IFNγ production. **E**-**H**. To determine the difference between stable and non-stable patient populations, concentrations of GM-CSF, TNFα, IL-17a, and IFNγ cytokine production was analyzed between the two groups. Each patient was run in triplicate, error bars ± SEM, *P < 0.05.

To further investigate an immune response to vaccination, patients’ immature dendritic cells were pulsed with the vaccine derived tumor lysate, maturated and then incubated with peripheral blood mononuclear cells (PBMCs) from each patient at different time points. Supernatants were harvested and assayed for cytokine elaboration. Following lysate stimulation, patient cells produced GM-CSF (Figure 4A), TNFα (Figure 4B), IL-17a (Figure 4C), and IFNγ (Figure 4D). PBMCs from patients were incubated with non-lysate pulsed DCs as a control and no cytokine response was observed. There was a statistically significant increase in elaboration of GM-CSF (Figure 4E) and TNFα (Figure 4F) between days 0 and 12 in the stable patient group. In addition, an increase of IL-17a was observed, (Figure 4G). However, it failed to reach statistical significance. No IFNγ difference was observed between groups (Figure 4H). Interestingly, there was no detectable IFNγ production by patient 8 throughout the trial (Figure 4D).

**Figure 5:**
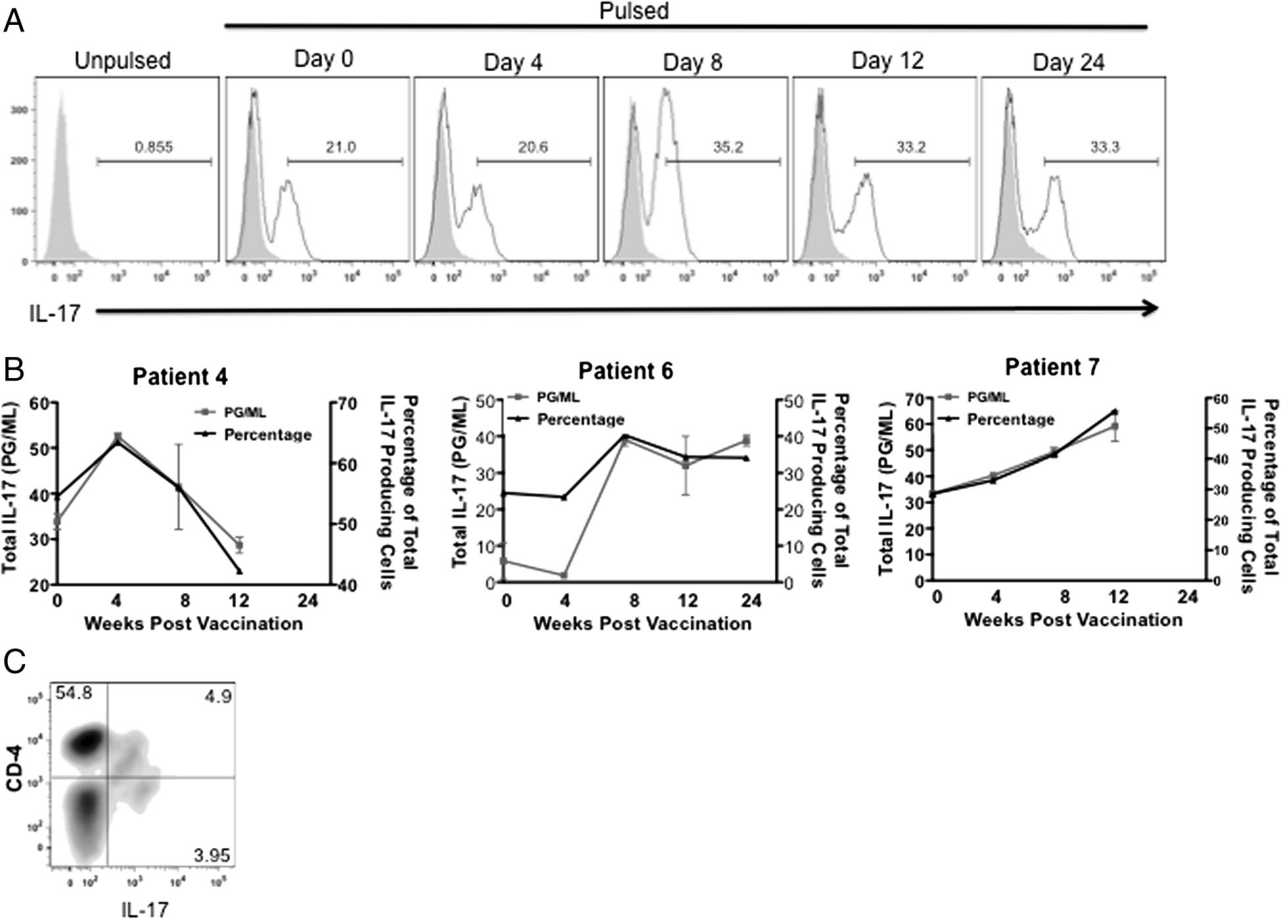
**CD4 T cell produce IL17.** Patients’ PBMCs collected on or days 4 through 24 post vaccination were stimulated with tumor lysate-pulsed immature dendritic cells and analyzed for **A**. total IL-17a production and **B** total secreted IL-17a plotted with percentages of IL-17a producing cells. **C**. CD4 T cells were analyzed for intracellular IL-17a production. Each patient was run in triplicate, error bars ± SEM, *P < 0.05.

CD4^+^ T cells were primarily known to be Th1, Th2, or Treg cells; however, more recently, a subset of IL-17a–secreting CD4^+^ T cells (Th17 cells) has been characterized [21,22]. Further studies have reported that MDSCs are able to modulate the induction of Th17 cells from CD4^+^ T cells, and also catalyze the differentiation of Foxp3^+^ regulatory T cells from monocyte-induced Th17 cells [23]. In this study, we observed an increase of IL-17a in the stable patients. Therefore, we repeated our experiment by harvesting cells 24 hours post stimulation and analyzed total intracellular IL-17a production. We observed the same trend as seen in total secreted IL-17a production (Figure 5A). Plotting percentages of total IL-17a producing cells against concentrations of secreted protein demonstrated the same trend of total IL-17a in patients over time (Figure 5B). To determine which T cell population is responsible for the production of IL-17a, we repeated the experiment. Using flow cytometry, by gating on the CD3^+^ population, we determined that IL-17a is produced by CD4^+^ T cells (Figure 5C).

Due to the lack of effective treatment for malignant brain tumors, extensive effort has been applied to the development of new immunotherapeutic approaches. This study represents the first-ever use of an allogeneic cell source for vaccine immunotherapy of individuals with these diseases. Numerous practical difficulties are encountered in personalized therapy using autologous material. Therefore, we sought to derive a novel highly immunogenic allogeneic cell line expressing high levels of multiple tumor antigens for use in a DC vaccine. In this phase I study, twelve patients were enrolled; two patients were unable to proceed with vaccine due to rapid disease progression following apheresis, one patient was ineligible following revision of diagnosis and one patient was unevaluable following treatment for a pre-existing surgical complication after only one vaccine dose. Eight patients ultimately received the vaccine generated by incubation of DCs with allogeneic glioma cell line GBM6-AD at dose levels of 5 million, 10 million and 15 million DCs. No DLTs were encountered. We are encouraged by the observations of partial response in one patient and prolonged survival achieved after protocol therapy in 2 other patients. The problem of vaccine feasibility in the face of tumor progression, especially after discontinuation of bevacizumab therapy, is significant given the very malignant nature of the tumors in our treatment subjects. This was especially evident in the patients who underwent apheresis, but progressed prior to initiation of vaccine therapy and patient 4, who had post-bevacizumab radiographic changes (data not shown) and eventual progression by our study criteria.

We sought to use immune biomarkers and cytokine profiling throughout the study to discern a correlation with clinical response. Using these markers, we separated our patients into stable and non-stable patient populations. This separation not only revealed a significant increase in MDSCs in the non-stable patient population (Figures 1A-C), but also revealed a significant increase in the absolute number of natural killer cells in both healthy donor and stable populations compared to non-stable patients (P = 0.02 and P = 0.04 respectfully) (Additional file 2: Figure S2). The reduced natural killer population in non-stable patients may result in reduced IFNγ production and decreased tumoricidal response. In addition, although not statistically significant, we observed an increased CD8^+^CTLA-4^+^ population in non-stable patients (data not shown), inferring that the checkpoint blockade inhibitor ipilimumab may be beneficial in this population.

CD4 T cells have recently been characterized to produce IL-17a [24], and it has been reported that the majority of IL-17a - producing effector cells produce IL-2 and TNFα, but fail to produce IFNγ or granzyme B [24,25]. In the present study, we saw a similar trend in one of our patients, patient 6, who was characterized as stable and failed to produce IFNγ. This patient did demonstrate a significant increase in IL-17a by week 8 that continued throughout his participation in the trial. The lack of IFNγ production may be due to the lack of CD4^+^ T cells. Throughout the study, this patient had an average 26% CD3^+^ CD4^+^ population, the lowest of any patient. Interestingly, from this low percentage of CD3^+^ CD4^+^ population, patient 6 maintained an average of 9.1% naïve CD4^+^ (CD4^+^CD27^+^CD45RA^+^CD62L^+^) T cell population, the lowest of all patients, but had an average of 52.4% memory (CD4^+^CD27^+^CD45RA^+^CD62L) CD4 T cells, the highest of all patients. Although this patient went off trial, he had the longest progression free survival (39.7 weeks) and survived for >100 weeks post vaccination.

## Conclusions

This study was designed to determine the safety of a novel allogeneic brain tumor vaccine. While we accomplished this goal, we also performed post-hoc analyses to determine the usefulness of phenotypic biomarkers and cytokine production to follow the efficacy of the vaccine in producing an immune response. We discovered a potential correlation of MDSC levels with response to vaccination. This is important because understanding the mechanism (s) allowing the disease to escape treatment will allow us to modify our therapy to better suit future patients. By measuring *in-vivo* phenotypic markers and cytokine responses *ex-vivo*, we were able to follow alloresponses to vaccine over time in the stable patient population. Such marker analyses will be important in evaluating the effectiveness of future vaccine trials and may be prognostically important for patients being considered for immunotherapy. While this study demonstrates safety in the use of a novel allogeneic vaccine, we show the potential importance of using specific biomarkers to evaluate and predict vaccine effectiveness. We do acknowledge that this study is underpowered. Therefore, based on our observations, our future study design will focus on the biomarkers reported in this manuscript. Despite the small patient number in this pilot study, we believe this is important information to carry forward to future brain tumor immunotherapy trials.

## Methods

### Patients and eligibility

The protocol outline can be obtained at http://clinicaltrials.gov/ct2/show/NCT01171469?term=dendritic+cell++brain&rank=1. The University of Minnesota institutional review board approved the study. Informed consent was obtained from patients 18 years of age and older and from the parents/legal guardians of patients less than 18 years. Patients with histopathologically confirmed glioblastoma multiforme (WHO Grade IV), ependymoma and medulloblastoma were eligible for this trial. Pediatric subjects were ages 0 through 17 years and adult subjects were 18 years and above. All patients were required to have received and failed standard-of-care therapy for their respective tumor and had a performance score of at least 60 based on the Lansky play performance scale for patients 0 – 15 years and a Karnofsky performance status of at least 60% for patients 16 years and older. Patients were required to have adequate baseline bone marrow, hepatic and renal function. All female patients of child bearing potential had a negative pregnancy test. Patients were excluded for uncontrolled intercurrent illness including, but not limited to, ongoing or active infection, symptomatic congestive heart failure, unstable angina pectoris, or psychiatric illness/social situations that would limit compliance with study requirements or if they were currently receiving any other investigational agents. Patients who had a history of immune system abnormalities such as hyperimmunity (e.g., autoimmune diseases) and hypoimmunity (e.g., myelodysplatic disorders, marrow failures, AIDS, ongoing pregnancy, transplant immuno-suppression) were also ineligible, as were patients with conditions that could potentially alter immune function (diabetes, renal failure, liver disease, severe nutritional depletion).

### Dendritic cell generation

At enrollment, each patient underwent leukapheresis to obtain peripheral blood mononuclear cells. The patient subjects were required to complete a donor questionnaire/screen comparable to current standard blood center collection practice, and standard tests for infectious diseases were performed. Infectious disease testing included screens for HIV and HCV (by nucleic acid testing), anti-HIV I/II, anti-HTLV I/II, anti-HBc Ab, HBsAg, anti-HCV, anti-CMV and *Treponema pallidum* (by serology). One standard-volume (e.g., 12–15 L) of non-mobilized apheresis product was enriched for monocytes using the Miltenyi Biotec CliniMACS® Cell Selection System and CD14 MicroBeads and reagent (Miltenyi Biotec, Bergisch Gladbach, Germany). This system typically yields ≥ 60% recovery and ≥ 90% purity. Approximately 8 × 10^8^ monocytes went into the culture system (polystyrene tissue culture flasks; 37°C at 5% CO_2_) at a concentration of 3–5 × 10^6^ cells/mL, GM-CSF (25 ng/mL) and IL-4 (40 ng/mL) were added in addition to fresh medium measuring one third of the volume of the initial culture, were added on day 3 and day 5 to derive immature dendritic cells (iDC). Cells were then maturated by adding GM-CSF (50 ng/ml), IL-1β25 ng/mL), TNF-α (50IY/μλ), Poly-IC (20 ng/ml), IFN-α (1500IY/μλ), IFN-γ (3,000 units /mL) and incubated at 37°C for 2 days. Mature DCs were harvested on day 7 and were cryopreserved and stored until they were incubated with tumor cell lysate at a later date. A DC yield of 25-30% was expected (approximately 2 × 10^8^ DCs). Analysis by flow cytometry (CD1a, CD14, CD16, CD83, CD86, HLA-DR) was used to characterize the final product. Mature DC purity was expected to be ≥70%. An initial viability assessment was performed on a pre-processing aliquot and on a sample drawn from the final product. Viability testing was performed by flow cytometry [e.g., 7-amino actinomycin D (AAD)]. A viability of ≥70% was expected on the final product.

### Vaccine development

We established an allogeneic brain tumor stem cell line (GBM6-AD) that was used as the source of tumor antigen based on the cell line’s high expression of tumor stem cell markers, tumor associated antigens, immunogenicity and rapid growth in culture [15]. The original brain tumor specimen was delivered on ice in a tube containing Neurobasal (NB) media (Invitrogen). Upon arrival to the laboratory, tumor tissue was transferred to NB supplemented with 50 ng/mL bFGF, 50 ng/mL EGF, 5 μg/mL gentamicin, and 0.9 μg/mL Fungizone under serum free conditions. The tissue was transferred to a Petri dish and minced using scalpels until the tissue particles were of a size that could be drawn in and out of a 10 ml pipet. The cell suspension was strained to remove any connective tissue. The cells were washed in PBS and once in Red Blood Cell Lysis solution. Cultures were grown in a cell culture incubator at 37°C, 5% O_2_, and 90% relative humidity in culture medium consisting of: DMEM/F12 w/ L-glutamine and sodium bicarbonate (Invitrogen), B27 supplement (0.5×) (Invitrogen), N2 supplement (0.5×) (Invitrogen), 20 ng/ml human EGF (Pepprotech), 20 ng/ml human FGF (Pepprotech), 1× non essential amino acids (Gibco), 1% penn/strep (Gibco), and 10 mM Hepes (Gibco) until confluent. To generate apoptotic bodies, adherent GBM6-AD cells were incubated between 44- 46°C in an incubator for 3 hours and returned to 37°C. After 24 hours, cells were harvested, washed, and irradiated (200 Gy) and cryopreserved in 10% DMSO. Apoptotic bodies were added to cultured iDCs and maturated as described above. Vaccine was stored at −150°C in liquid nitrogen vapor. All final product was required to pass lot release criteria prior to use (Table 2).

Each patient received a single subcutaneous injection of vaccine (see Table 1) given in alternating suprascapular areas after topical imiquimod pre-administration at the vaccine injection site. Imiquimod was then administered again to the injection site at 24 hours post-vaccine. Imiquimod is marketed as 5% Aldara cream in 250 mg packets, providing a total dose of 12.5 mg per packet, which is sufficient to cover 20 square centimeters. The contents of one half packet were applied as a thin film to cover approximately 10 square centimeters of skin in the area of the vaccination. The imiquimod was rubbed in well using a gloved finger. The leftover imiquimod was disposed of and a new packet used for each application. Patients were observed for 30 minutes after each injection for immediate systemic or injection site adverse events.

**Table 2:** Median age of patients 48.5 (3–71)

**Patient ID**	**Age**	**Gender**	**Performance status**	**Diagnoses**	**Number of vaccinations**	**Time to progression (Weeks)**	**Survival (Weeks)**	**Maximum related toxicity/grade**	**Response status**
Patient 1	71	Male	80	GBM	2	**4.2**	34.6	ISR/1	Non-stable
Patient 2	61	Male	90	GBM	4	8	12.4		Non-stable
Patient 3	42	Female	80	GBM	I	Not evaluable	N/A		Non-stable
Patient 4	55	Female	90	GBM	5	13.0	64.8	ISR/ 1	Stable
Patient 5	17	Male	70	GBM	10	30.6	53.3	lSR/1	Stable
Patient 6	63	Male	90	GBM	9	39.7	>92	ISR/1	Stable
Patient 7	3	Female	60	Epend	6	20.0	31.4	Fat/i	
*ISR/3*	Stable								
Patient 8	24	Female	90	Med	5	8.0	>92		Non-stable
Patient 9	53	Male	80	GBM	0	N/A	N/A		Non-stable
Patient 10	13	Female	90	PNET	0	N/A	N/A		Non-stable
Patient 11	49	Male	80	GBM	0	N/A	N/A		Non-stable
Patient 12	48	Male	80	GBM	0	N/A	N/A		Non-stable

Male: Female 4:4. prior treatment regimes median (range) 1.5 (1–4). Performance Status (KPSansky), GBM Glioblastoma (WHO Grade IV), Epend = Ependymoma. Med = Medloblastoma, PNRT = primitive neuroectodemial tumor. ISR = Injection site skin reaction. Fat = Fatigue. Survival is measured in weeks post treatment Patients 9–12 failed trial prior to vaccination.

### Study design

The study protocol dictated vaccine administration at day 0 and every two weeks for the first 8 weeks. Thereafter, re-vaccination was administered every 4 weeks up to an additional 10 vaccinations for patients without dose limiting toxicity (DLT) or disease progression defined by greater than 25% tumor growth on MRI. This study used an accelerated dose escalation design (1 subject per level), after which a traditional phase I design (3 subjects per level) was implemented. DLT was defined as Grade 3 or greater treatment related toxicity in association with the first two vaccinations. No DLT was encountered. Escalation to the next dose level did not occur until the patient (s) in the previous cohort were at least 4 weeks post the initial vaccination. Patients 1 and 2 received 5 million and 10 million DCs, respectively. Patients 3 through 8 received 15 million DCs.

### Toxicity assessment and disease evaluation

Toxicity monitoring included medical history, physical examination, complete blood counts and serum chemistries prior to each vaccine episode. DLT was defined as Grade 3 or greater treatment related toxicity. Toxicity and adverse events were classified according to NCI’s Common Terminology Criteria for Adverse Events V 3.0 (CTCAE). Skin reactions of Grade 3 were not defined as a DLT.

Subjects were evaluated using 3 Tesla MRI to determine tumor size prior to treatment to obtain a baseline measurement. Subjects were then re-evaluated by 3 Tesla MRI at monthly intervals through the first 3 months of treatment and once every 3 months thereafter.

### Immune assessment

Blood was drawn from patients 2 weeks prior to vaccination, at vaccination, and on weeks 4, 8, 12, and 24. Purified peripheral blood mononuclear cells were analyzed for cytokine analysis, whole blood was analyzed by flow cytometry for MDSC ((Lineage-negative), FITC (CD3, CD14, CD16, CD19, CD20, CD56) CD33-APC, HLA-DR-PerCp)), monocytic MDSC (CD14-APC, HLA-DR-PerCp), granulocytic MDSC (CD15-FITC, CD14-APC), CD8 central and effector memory (CD45RO-PE, CD8-PerCp, CD62L-APC, CCR7-FITC) and CD4 central and effector memory (CD45RO-PE, CD4-PerCp, CD62L-APC, CCR7-FITC) populations. 5 × 10^5^ patient immature dendritic cells were suspended in 1 ml of AIM V media (Life Technologies) and pulsed with 10 μg of tumor lysate derived from the vaccine cell line and maturated with human recombinant GM-CSF (50 ng/ml), IL-4 (40 ng/ml), IL-1β (25 ng/mL), TNFα (50 ng/mL), Poly-IC (20 ng/mL), IFN-α (1500IY/μλ), and IFNγ (3,000 units/mL). Cultured DCs were washed twice and 1 × 10^6^ PBMCs isolated from each patient at weeks 0, 4, 8, 12, and 24 were added to DCs and incubated for 48 h. Supernatants (50 μl) was removed and analyzed for IFNγ, GM-CSF, TNFα, and IL-17a. To validate cytokine production, in separate wells, PBMCs were incubated with maturated DCs for 24 hrs, Golgi Stop (BD Biosciences) at a final dilution of 4 μl/6 ml was added to wells. PBMCs were labeled with CD3-APC, CD4-FITC, CD8-PE, fixed and permiabilized (BD Biosciences) and stained with IL-17a eFluor450. Cells were washed and analyzed by flow cytometry. In both sets of experiments, non-tumor lysate pulsed DCs were used as a control.

### Hierarchical clustering of immune phenotypes

Conversion of immune phenotypes to cells per microliter and hierarchical clustering of immune phenotypes were performed. In this study, the phenotypic values were normalized by dividing the value by the mean value of healthy volunteers. The immune phenotype ratios were imported into Partek Genomics Suite 6.6 software (Partek Inc., St. Louis, MO) and log-transformed for hierarchical clustering. Hierarchical analysis was performed by unsupervised agglomerative Euclidean average linkage clustering [21].

### Assessment and statistical analysis

In this study, we established four response categories based on a modification of the criteria proposed by MacDonald et al. [26]. Complete responses (CR) are those in which there is a disappearance of all enhancing tumor or tumor mass on consecutive MRI scans. Patients must be off steroids, and neurologically stable or improved. Partial responses (PR) are those in which there is a ≥ 50% reduction in the size of the enhancing tumor or tumor mass on consecutive MRI scans. In addition, the patient must be neurologically stable or improved. A progressive disease (PD) state is one in which there is ≥ 25% increase in the size of the enhancing tumor or tumor mass on MRI scans, or the patient is neurologically worse, and steroids are stable or increased. A stable disease (SD) state is all other situations. If patients initially exhibit tumor regression or a stable response for a period of time followed by progressive disease, this would be viewed as a relapse.

Statistical comparisons were made by comparisons using a 2-tailed *t-test*. All tests were performed with Prism 4 software (Graph Pad Software, Inc). P values <0.05 were considered significant.

## Competing interest

The authors declare that they have no competing interest.

## Authors’ contributions

MRO: Contributed on all immunology, vaccine production, writing of the manuscript and figures. CLM: supervised the clinical trial as primary investigator and provided direct patient care to all study participants. He contributed to the writing of the manuscript. WL: Revisions of manuscript, optimization of DC protocol and IND development. DHM: vaccine development, GLP manufacturing and IND development. SH: phase I protocol, IND development and patient care. TD: nursing care. All authors read and approved the final manuscript.

## Supplementary Material

Additional file 1: Figure S1 Effector and memory T cell populations responded to vaccination in the stable patient population. Patients’ whole blood was stained with antibodies to measure A. CD4 central memory, B. CD8 central memory, C. CD4 effector memory and D. CD8 central memory populations. Patients are individually plotted in plots A-D. E-H. Percentages from stable and non-stable patient populations were combined to analyze various cell phenotypes. Each patient was run in triplicate, error bars ± SEM, *P < 0.05. Click here for file

Additional file 2: Figure S2 The stable patient population has a higher natural killer population. Patients’ whole blood was stained with antibodies to analyze natural killer populations from patients prior to vaccination. Each patient was run in triplicate, error bars ± SEM, *P < 0.05. Click here for file
