# Supplementary figures and images for: Vaccination with dendritic cells loaded with allogeneic brain tumor cells for recurrent malignant brain tumors induces a CD4+IL17+ response
Source: J Immunother Cancer. 2014 Feb 18;2:4. doi: 10.1186/2051-1426-2-4 (PMC4019901; doi:10.1186/2051-1426-2-4)

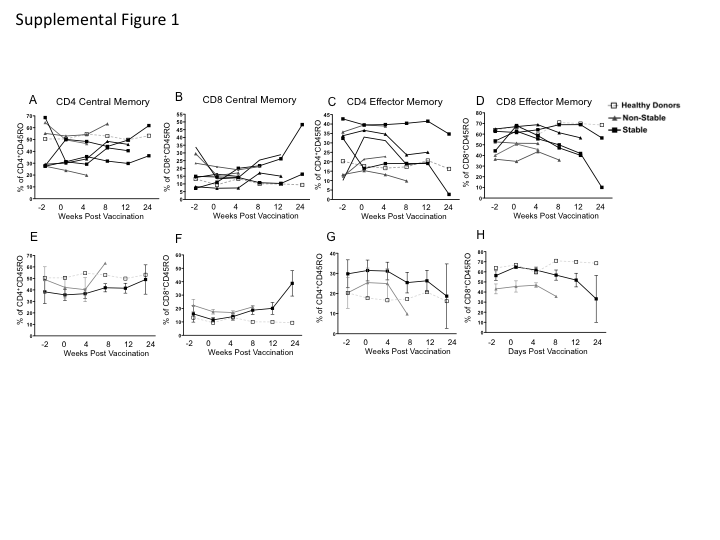

Supplement: Additional file 1: Figure S1 — Effector and memory T cell populations responded to vaccination in the stable patient population. Patients’ whole blood was stained with antibodies to measure A. CD4 central memory, B. CD8 central memory, C. CD4 effector memory and D. CD8 central memory populations. Patients are individually plotted in plots A-D. E-H. Percentages from stable and non-stable patient populations were combined to analyze various cell phenotypes. Each patient was run in triplicate, error bars ± SEM, *P < 0.05. [file 2051-1426-2-4-S1.tiff]

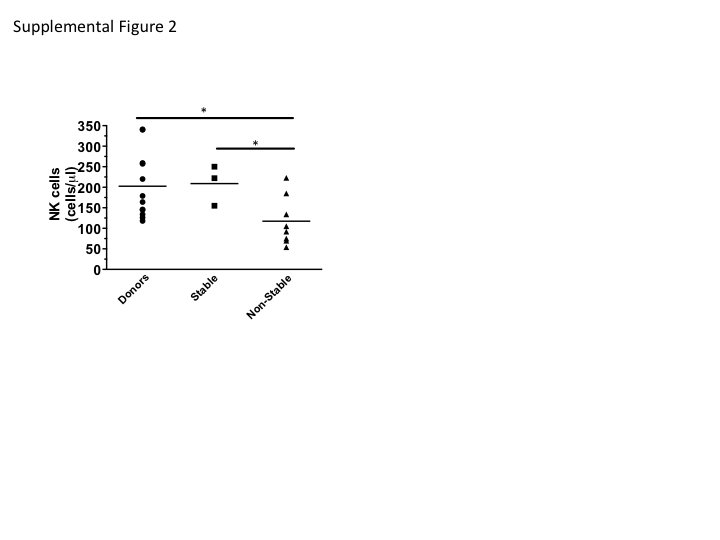

Supplement: Additional file 2: Figure S2 — The stable patient population has a higher natural killer population. Patients’ whole blood was stained with antibodies to analyze natural killer populations from patients prior to vaccination. Each patient was run in triplicate, error bars ± SEM, *P < 0.05. [file 2051-1426-2-4-S2.tiff]
